# Bilingualism and “brain reserve” in subregions of the hippocampal formation

**DOI:** 10.1007/s11357-025-01639-0

**Published:** 2025-04-08

**Authors:** Katharina Peitz, Nora Bittner, Stefan Heim, Svenja Caspers

**Affiliations:** 1 Institute for Anatomy I, Medical Faculty & University Hospital Düsseldorf, Heinrich Heine University, Düsseldorf, Germany; 2 Institute of Neuroscience and Medicine (INM-1), Research Centre Jülich, Jülich, Germany; 3 Department of Psychiatry, Psychotherapy and Psychosomatics, Medical Faculty, RWTH Aachen University, Aachen, Germany

**Keywords:** Aging, Bilingualism, Hippocampus, Subicular complex, Gray matter volume, Brain reserve

## Abstract

With aging, the hippocampal formation shows variable structural atrophy, which is associated with a decline in cognitive performance. Bilingualism is related to higher hippocampal gray matter volume (GMV), potentially representing a form of brain reserve in aging. However, the differential influence of bilingualism on hippocampal subregions remains unclear. Thus, we investigated GMV differences and differences in age-GMV relationships between mono- and bilinguals in the hippocampal formation and its subregions, hippocampus proper and subicular complex. We included 661 adults aged 19 to 85 years (257 monolinguals, 404 sequential bilinguals, predominantly native German speakers with variable second language background) from the population-based 1000BRAINS cohort. GMV differences in mono- vs. bilinguals were assessed for six regions of interest (hippocampal formation, hippocampus proper, and subicular complex; each left and right) using analyses of covariance. Effects of bilingualism on age-GMV relationships were investigated via moderation analyses. We found higher GMV in bilinguals in the bilateral subicular complex, while only a trend towards this effect existed for the hippocampal formation. Moderation analyses revealed similar age-GMV relationships between mono- and bilinguals for all regions of interest. Higher GMV in bilinguals’ hippocampal formation seems specifically attributable to the subicular complex rather than the hippocampus proper. With similar age-GMV relationships for mono- and bilinguals, bilingual brain reserve in the subicular complex may persist over time. This may be particularly beneficial since subicular atrophy has previously been associated with higher risk for dementia. Altogether, a differential impact of bilingualism on hippocampal subregions has been demonstrated.

**Supplementary Information:**

The online version contains supplementary material available at 10.1007/s11357-025-01639-0.

## Introduction

The hippocampal formation is considered one of the brain regions most sensitive to the inter-individually variable structural atrophy that comes with aging (e.g., [1, 2]; for review, cf. [3, 4]). With its relevance for memory and learning and being essentially involved in spatial processing, emotional control, attention, and language-related functions such as vocabulary acquisition [5–8], hippocampal atrophy may play a crucial role in age-related cognitive impairment [9]. Regarding neurodegenerative diseases, a faster rate of hippocampal volume decline is considered a reliable marker for risk and progression of dementias such as Alzheimer’s disease [3, 10–12].

Within the hippocampal formation, two subregions are distinguished along a dorsal/ventral axis, each consisting of cytoarchitectonically distinct subfields arranged along a medial/lateral axis: the hippocampus proper, comprising fascia dentata and cornu ammonis (CA; subdivided into CA1–CA4; fascia dentata and CA4 form the macroscopically visible dentate gyrus (DG)), and the subicular complex, consisting of prosubiculum, subiculum, presubiculum, and parasubiculum [13].

Hippocampal subregions show a differential susceptibility to age-related structural atrophy. While a relationship between age and structural decline has been reported for both, hippocampus proper subfields and the subicular complex, subicular cortices seem to display an association between higher age and smaller volumes more consistently than other subfields [14, 15]. Atrophy of the subiculum and presubiculum may actually be the earliest anatomical marker of Alzheimer’s disease within the hippocampal formation [16], and, importantly, reduced subicular volume has previously been associated with poorer cognition and a higher risk of dementia [17].

Two mechanisms have been proposed to potentially counteract the effects of aging: cognitive reserve and brain reserve. Cognitive reserve pertains to differences in cognitive processing with respect to efficiency, capacity, and flexibility of neural networks, putatively resulting in a differential ability for compensation when confronted with age-related structural decline. Brain reserve, on the other hand, refers to structural features of the human brain such as gray matter volume (GMV), cortical thickness, and white matter integrity [18, 19]. Individuals with higher brain reserve are assumed to be able to tolerate greater structural atrophy before falling under a given threshold below which cognitive deficits would become apparent [18–20].

Plastic structural adaptations of the human brain to behavior and experiences may potentially alter individual brain reserve [19]. Here, the hippocampal formation has shown remarkable malleability. For taxi drivers in London, for example, years of navigation experience correlate with hippocampal gray matter volume, likely reflecting large spatial knowledge [21, 22]. Even in older individuals, behavioral or physical interventions appear to increase hippocampal volume (for review, cf. [23–25]). One factor that has been found to have an impact on hippocampal volume is bilingualism.^1^

Bilingualism is thought to mitigate effects of aging through both, higher cognitive reserve and brain reserve [20]. It imposes specific cognitive demands onto the human brain. As several languages seem to be simultaneously active in the bilingual mind [26, 27], bilingualism is thought to require constant conflict monitoring, conflict resolving, interference suppression, and correct language switching when engaging in communication [28–30]. Hence, bilingualism may represent a form of cognitive exercise that appears to result in differences in cognitive processing when compared to monolinguals [31]. These differences seem to induce cognitive advantages, e.g., with respect to domain-general cognitive functions and memory (for reviews, cf. [32–35]). As the cognitive advantages in bilinguals appear especially evident in the older adult population [36], they may reflect a form of cognitive reserve in bilinguals. This might explain why bilingualism seems to delay age-related cognitive decline not only in healthy older adults (e.g., [37–40]; but see also Antón et al. [41] and Paap and Greenberg [42]), but also with respect to neurodegenerative diseases (e.g., the clinical onset of dementia seems delayed by 4 to 5 years in bilinguals [43–45]; meta-analyses in [34, 46]).

The cognitive challenges of bilingualism result in structural adaptations of the human brain. Bilingualism has been related to higher gray matter volume, higher cortical thickness, and higher white matter integrity as proxies of brain reserve in brain regions relevant for language and domain-general control (for reviews, cf. e.g. [47–49]). Furthermore, the relationship between age and brain structure may differ between mono- and bilinguals [50–52], and longitudinal studies have indeed shown that bilingualism might alter trajectories of structural brain parameters over time even in long-term bilinguals not undergoing any current language training [37, 53, 54]. However, regarding structural adaptations of the hippocampal formation to bilingualism, previous studies have yielded variable findings.^2^ In training studies with young adults, significant GMV increases have been found for the right [55] and bilateral [56] hippocampal formation after 2.5 and 3 months of language training, respectively. In healthy older adults, a recent cross-sectional study found an association between greater bilingual engagement and higher GMV in the hippocampal formation [57]. For patients with mild cognitive impairment, a non-linear effect of bilingual language entropy (describing the diversity of language use, ranging from 0 (single language use) to 1 (perfectly balanced use of languages)) on hippocampal volume has been reported, with the highest hippocampal GMV apparent for mid-range language entropy [58]. On the other hand, for healthy adults, Olsen et al. [59] as well as Torres et al. [60] found no GMV differences between mono- and bilinguals within the bilateral hippocampal formation. Regarding the relationship between age and hippocampal GMV, the left hippocampal formation has been reported to be more vulnerable to aging in monolinguals when compared to bilinguals, since evidence for a higher rate of age-related decline in monolinguals has been found for this region [52]. Interestingly, this effect was present not only for unimodal bilinguals (with abilities in spoken languages only), but also for bimodal bilinguals (i.e., individuals using a spoken and a signed language), putatively indicating common adaptations to bilingualism irrespective of language modality [51]. Furthermore, a longitudinal study found significant reshaping of the bilateral hippocampal formation across a time interval of 3 years in bilinguals who were highly immersed in their second language environment, which has been interpreted as a consequence of continuous use of and exposure to the respective second language [54]. At the same time, though, Olsen et al. [59] reported no difference in the relationship between hippocampal GMV and age for mono- vs. bilinguals. In general, variable findings in bilingualism literature might be explained by differences in sample and methodology [61, 62]. Additionally, previous studies investigating the impact of bilingualism on the hippocampal formation were based on rather small to medium-sized samples ([54], *n *= 9; [56], *n *= 31; [58], *n *= 40; [59], *n *= 42; [51], *n *= 43; [52], *n *= 46; [57], *n *= 48; [55], *n *= 56), the study from Torres et al. [60] being an exception (*n *= 214). Furthermore, previous studies have evaluated the hippocampal formation as one single structure, while the differential influence of bilingualism on its subregions remains unclear.

Hence, we aimed to investigate GMV in mono- and bilinguals not only in the bilateral hippocampal formation, but also specifically within the bilateral hippocampus proper and subicular complex in a large, population-based cohort. As bilingualism is increasingly viewed as a continuum rather than just a dichotomous variable [63], we performed not only group comparisons, but assessed the impact of factors modulating the bilingual experience, such as age of acquisition (AoA), level of proficiency (LoP), bilingual engagement, and number of actively spoken languages, on hippocampal GMV as well. Next, we evaluated the influence of bilingualism on the relationship between age and GMV in the left and right hippocampal formation and the respective subregions. To set a focus on brain structure within older adults, all analyses were analogously performed within a subsample including only individuals ≥ 55 years old.

## Methods

### Participants

The participants included in the present study were drawn from the population-based 1000BRAINS cohort [64]. 1000BRAINS is a cohort study designed to examine inter-individual variability during brain aging in healthy adults. Participants for 1000BRAINS were recruited from two studies conducted in the German Ruhr area, the Heinz-Nixdorf Recall (HNR) study, and the consecutive HNR MultiGeneration study, which investigate risk factors for atherosclerosis, myocardial infarction, and cardiac death [65, 66]. Due to the population-based nature of 1000BRAINS, exclusion from the study was based solely on eligibility for magnetic resonance imaging: contraindications were the presence of coronary artery stents, cardiac pacemakers, surgical implants or prostheses in the trunk or head, claustrophobia, past neurosurgery, tattoos or permanent make-up on the head, and, as a relative contraindication, dental implants and bridges (see [64]). All participants provided written informed consent before participating in 1000BRAINS. The study protocol was approved by the local Ethics Committee of the University of Essen, Germany.

Out of the 1314 individuals included in 1000BRAINS, participants with structural MRI data of sufficient quality as well as complete Language Experience and Proficiency Questionnaire data (LEAP-Q) [67] were eligible for the present investigation (*n *= 869). From these, left-handed individuals (*n *= 18, Laterality Quotient <−60 as obtained with the Edinburgh Handedness Inventory [68]) and participants who did not provide any data with respect to handedness (*n *= 1) were excluded. An exclusion criterion derived from LEAP-Q data was simultaneous bilingualism (*n *= 17; while simultaneous bilinguals acquire two languages from birth, usually in form of a naturalistic language experience, sequential bilinguals start learning a second language later in life, often in a classroom setting [69]. These diverging bilingual experiences appear to have distinct repercussions in brain structure: simultaneous bilinguals show smaller structural differences than sequential bilinguals when compared to monolinguals ([70]; for review, cf. [71, 72]), and differences between simultaneous and sequential bilinguals seem to remain existent during adulthood and when learning additional languages [69]). Furthermore, individuals who reported developmental first language deficiencies in any modality (speaking, comprehending, reading, writing) were excluded from the sample (*n *= 70). After exclusion of eight participants due to outlier correction (GMV in hippocampal formation, hippocampus proper, and/or subicular complex exceeding three standard deviations from the mean), the total sample of the present study consisted of 661 participants (257 monolinguals and 404 bilinguals; Table 1). The older subsample, including only participants older than 55 years, comprised 470 individuals (238 monolinguals and 232 bilinguals; Table 1).

**Table 1:** Study sample: demographic characteristics

	Total sample (*n* = 661)		Older subsample (participants ≥ 55 y, *n* = 470)	
	Monolinguals (*n* = 257)	Bilinguals (*n* = 404)	Monolinguals (*n* = 238)	Bilinguals (*n* = 232)
Sex				
% Female	49.4	41.6	47.5	38.4
% Male	50.6	58.4	52.5	61.6
Age [years]				
Mean (SD)	67.1 (9.0)	55.6 (14.0)	68.8 (6.8)	65.6 (6.5)
Minimum	27.9	18.5	56.2	55.1
Maximum	85.4	82.5	85.4	82.5
Education level (SD)	5.8 (1.7)	7.6 (1.7)	5.8 (1.7)	7.5 (1.9)
AoA (SD) [years]	-	12.9 (6.8)	-	14.1 (8.4)
LoP (SD)	-	9.9 (3.1)	-	9.1 (3.0)
BiE (SD)	-	2.2 (0.9)	-	2.1 (0.9)
NoL (SD)	1 (0)	2.8 (0.8)	1 (0)	2.7 (0.8)

Group characteristics for monolinguals and bilinguals for the total sample (*n* = 661) as well as for the subsample including only participants ≥ 55 years old (*n* = 470). For AoA, LoP, and BiE, the values of the second language for which the respective individual had reported highest proficiency were used. LoP values ranged between 0 (none) and 16 (maximum proficiency), while BiE values ranged between 0 (none) and 4 (maximum bilingual engagement)*SD* standard deviation, *y* years, *AoA* age of acquisition, *LoP* level of proficiency, *BiE* bilingual engagement, *NoL* number of actively spoken languages

### Assessment of bilingualism

To obtain the second language status of each participant, LEAP-Q data [67] were acquired. The LEAP-Q is a validated and reliable self-assessment questionnaire that can be used to determine language profiles in bilingual and multilingual individuals with respect to AoA, manner of acquisition, LoP, bilingual engagement, and immersion in a bilingual environment. For the current study, individuals who could presently speak, understand, read, and/or write in more than one language were classified as bilinguals. Correspondingly, participants who reported no or lost second language abilities were classified as monolinguals. All monolinguals within the present study spoke German only, while language backgrounds for bilinguals (all but twelve were native German speakers) are presented in Table 2.

**Table 2:** Languages spoken among bilinguals included in the present study

	Total sample	Older subsample
Language	Bilinguals (*n* = 404) with language abilities [%]	Bilinguals (*n* = 232) with language abilities [%]
German	100.00	100.00
English	98.02	96.55
French	40.10	40.95
Spanish	13.61	9.48
Latin	7.92	6.90
Italian	6.68	6.03
Russian	3.47	4.74
Dutch	3.47	4.31
Polish	2.23	2.16
Other	5.20	4.74

Distribution of bilinguals who reported language abilities for the respective language in percent. “Other” includes Ancient Greek, Arabic, Chinese (not further specified), Czech, Danish, Finnish, Greek, Indonesian, Japanese, Malayan, Portuguese, Serbian, Swedish, and Turkish (at least one and up to three participants of the total sample reported language abilities for these languages, respectively). Dialects were not considered within the present study, since only one participant reported language abilities for a dialect, and this participant had to be excluded from the sample

Second languages of an individual were ranked with respect to self-reported proficiency in the respective language. Subsequently, AoA and bilingual engagement values of the non-native language for which the respective bilingual had reported highest proficiency were used for the investigation of the impact of bilingualism-specific parameters on hippocampal GMV within bilinguals. Mean values for these variables within the present sample are reported in Table 1.

### MRI data

#### Data acquisition

Magnetic resonance imaging data were obtained on a 3 T Siemens Tim-TRIO MR scanner (Erlangen, Germany). 3D high-resolution T1-weighted magnetization-prepared rapid acquisition gradient-echo (MPRAGE) scans were acquired for each subject as part of an imaging protocol (for further details, see [64]) using a 32-channel head coil (176 slices, slice thickness = 1 mm, repetition time = 2250 ms, echo time = 3.03 ms, field of view = 256 × 256 mm^2^, flip angle = 9°, voxel resolution = 1 × 1 × 1 mm^3^).

#### Image processing

To conduct region-based morphometric analyses, magnetic resonance imaging data were processed using the Computational Anatomy Toolbox (CAT) version 12.8_r1871 [73] in SPM12 (https://www.fil.ion.ucl.ac.uk/spm/). Preprocessing consisted of three major steps: (i) tissue segmentation (including denoising, SPM’s standard “unified segmentation” [74], skull-stripping, and segmentation into gray matter, white matter, and cerebrospinal fluid), (ii) spatial registration, with individual images being registered to the ICBM 2009c Nonlinear Asymmetric space (MNI152 NLin2009cAsym, i.e., MNI space; https://nist.mni.mcgill.ca/icbm-152-nonlinear-atlases-2009/), and (iii) extraction of regional GMV based on the Jülich-Brain atlas [75] as implemented in CAT [73].

**Fig. 1:**
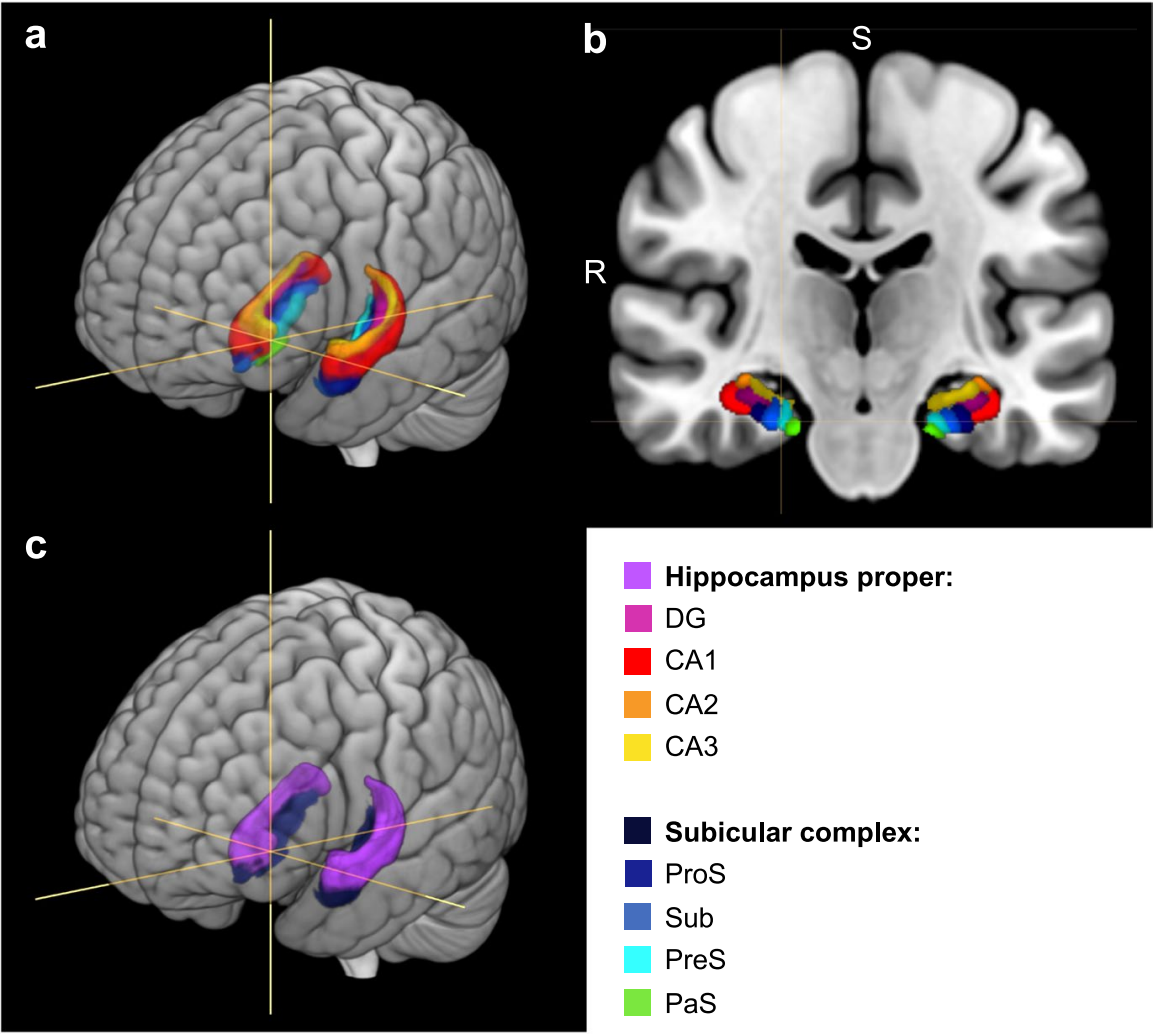
Subregions of the hippocampal formation based on the cytoarchitectonic probabilistic Jülich-Brain atlas [75] overlayed on an MNI template on a rendered brain (**a**) and in coronal view (**b**). Dentate gyrus (DG) and cornu ammonis (CA; including CA1, CA2, and CA3) formed the hippocampus proper (see **c**); the subicular complex consisted of prosubiculum (ProS), subiculum (Sub), presubiculum (PreS), and parasubiculum (PaS). R, right; S, superior

For each subject, GMV was extracted for the subregions of the hippocampal formation as cytoarchitectonically defined within the Jülich-Brain atlas [75] (hippocampus proper: DG and CA1–CA3; subicular complex: prosubiculum, subiculum, presubiculum, and parasubiculum [13] (see Fig. 1). Cytoarchitectonic maps can be found on EBRAINS (https://www.ebrains.eu) [76]. Total hippocampus proper and subicular complex volumes were obtained for each hemisphere by summing up GMV values of the associated cytoarchitectonic areas, while GMV for the left and right hippocampal formation was calculated as the sum of the respective hippocampus proper and subicular complex GMV values.

### Statistical analyses

Statistical analysis of extracted GMV data was conducted using the IBM Statistical Package for Social Sciences (SPSS), version 29.0.1 (https://www.ibm.com/docs/en/spss-statistics/29.0.0). Moderation analyses were performed using PROCESS [77] within SPSS.

#### Estimation of a linear vs. quadratic age-GMV relationship

We planned to conduct three types of analyses: (i) analyses of covariance (ANCOVAs), to assess GMV differences between mono- and bilinguals, (ii) regression analyses, to evaluate the impact of factors modulating the bilingual experience such as AoA, LoP, bilingual engagement, and number of actively spoken languages on hippocampal GMV in bilinguals, and (iii) moderation analyses, to see whether the relationship between age and GMV would differ between the two language groups. Age was to be included as covariate of no interest (ANCOVAs, regression analyses) or as predictor (moderation analyses) in the models. ANCOVAs, regression analyses, and moderation analyses are based on linear regression models. Thus, a linear relationship between covariate/predictor (i.e., age) and dependent variable (in this case, GMV) is assumed. However, a quadratic relationship between age and GMV within the hippocampal formation has been described previously, with evidence for an acceleration of volumetric decline from 55 to 60 years of age onwards ([78], for a meta-analysis, see [79]). Thus, we conducted hierarchical regression analyses to test whether a quadratic model for the relationship between age and hippocampal GMV would provide a better fit to our data than a linear one. To this end, we built two subsequent regression models for each ROI. GMV of the respective ROI was the dependent variable in each of the models. In the first model, *age* was submitted as sole predictor, corresponding to a linear estimation of the age-GMV relationship. In the second model, *age*^*2*^ was included as additional predictor, hence describing a quadratic relationship between age and GMV. If the inclusion of *age*^*2*^ as a predictor improved the model significantly, the quadratic model for the relationship between age and GMV was to be incorporated into subsequent analyses of the respective ROIs instead of the default linear model. All analyses were performed for the total sample as well as for the older subsample including only participants older than 55 years. For the sake of completeness, the analyses were additionally conducted for the younger subsample comprising only participants < 55 years old.

Based on the results that were obtained from hierarchical regression analyses (see Supplementary Tables 1–3), the relationship between age and GMV in subsequent analyses was modeled as follows: Within the total sample, analyses for the bilateral hippocampal formation and hippocampus proper were based on a quadratic relationship between GMV and age, while analyses for the bilateral subicular complex were based on a linear age-GMV relationship. For the older as well as younger subsamples, analyses for all ROIs were based on a linear age-GMV relationship.

#### ANCOVA models

To investigate GMV differences between mono- and bilinguals in subregions of the hippocampal formation, analyses of covariance (ANCOVAs) were conducted separately for each region of interest (ROI) (left/right hippocampal formation, left/right hippocampus proper, and left/right subicular complex). The respective GMV values were treated as dependent variables, while language group (monolinguals/bilinguals) served as between-subject factor. *Age* (and, for analyses of the bilateral hippocampal formation and hippocampus proper within the total sample, also *age*^*2*^), sex,^3^ education (as evaluated with the International Standard Classification of Education [80]), and intracranial volume (ICV) were included as covariates of no interest to control for potential confounding effects on GMV in all of the analyses.

ANCOVA models were performed for the total sample of 661 participants. However, below the age of 55 years, the number of mono- and bilingual participants was rather imbalanced. Therefore, to specifically investigate effects of bilingualism within older adults with a more homogeneous distribution of mono- and bilinguals, corresponding analyses were subsequently also performed for the subsample including only participants ≥ 55 years of age (238 monolinguals, 232 bilinguals; Table 1), the only adjustment being that *age*^*2*^ was not included as a covariate in analyses of the hippocampal formation and hippocampus proper to correspond to a linear model of the relationship between age and GMV (cf. Supplementary Table 2).

#### Regression analyses

Regression analyses were performed to study the impact of factors modulating the bilingual experience, such as AoA, LoP, bilingual engagement, and number of languages actively spoken, on GMV in the bilateral hippocampal formation and its subregions. By definition, these parameters were available for bilinguals only. Therefore, monolinguals were excluded from the analyses. For each ROI, separate analyses were conducted with GMV as dependent variable within (i) bilinguals of the total sample, (ii) bilinguals ≥ 55 years of age, and (iii) bilinguals < 55 years of age. Variables of interest (AoA, LoP, bilingual engagement, number of actively spoken languages) and covariates of no interest (*age* (and, for analyses of the bilateral hippocampal formation and hippocampus proper within the total sample, also *age*^*2*^), sex, education, ICV) served as predictors in all models.

While analyses of the total sample and the older subsample complemented group comparisons between mono- and bilinguals via ANCOVA analyses, regression analyses within younger bilinguals were exploratory to see whether relationships between GMV and factors modulating the bilingual experience would emerge in this age group.

#### Moderation analyses

To evaluate the effect of bilingualism on the relationship between age and GMV, moderation analyses were conducted for the total sample as well as for the older subsample. *Age* served as predictor (as well as *age*^*2*^ for analyses of the bilateral hippocampal formation and hippocampus proper within the total sample), GMV as dependent variable, and language group as moderator (bilinguals set to 0, monolinguals to 1). Sex, education, and ICV were included as covariates of no interest in all of the analyses.

### Post-hoc analyses: interaction of language group × sex

While only small effects of sex were present for left-hemispheric ROIs (and almost exclusively within the older subsample), a highly stable effect of sex was observed for the right subicular complex across almost all analyses (cf. Supplementary Tables 4–10). Thus, post-hoc analyses to investigate a potential interaction effect for language group × sex within this ROI seemed warranted. To this end, ANCOVA models as described in the “ANCOVA models” section, modified by treating sex as between-subject factor rather than covariate and by including the interaction term language group × sex, were performed for the right subicular complex within the total sample as well as the older subsample (as dichotomous group comparisons for mono- vs. bilinguals had previously been conducted for these two samples). For the sake of completeness, corresponding analyses were performed for the remaining ROIs.

## Results

### ANCOVA models

**Fig. 2:**
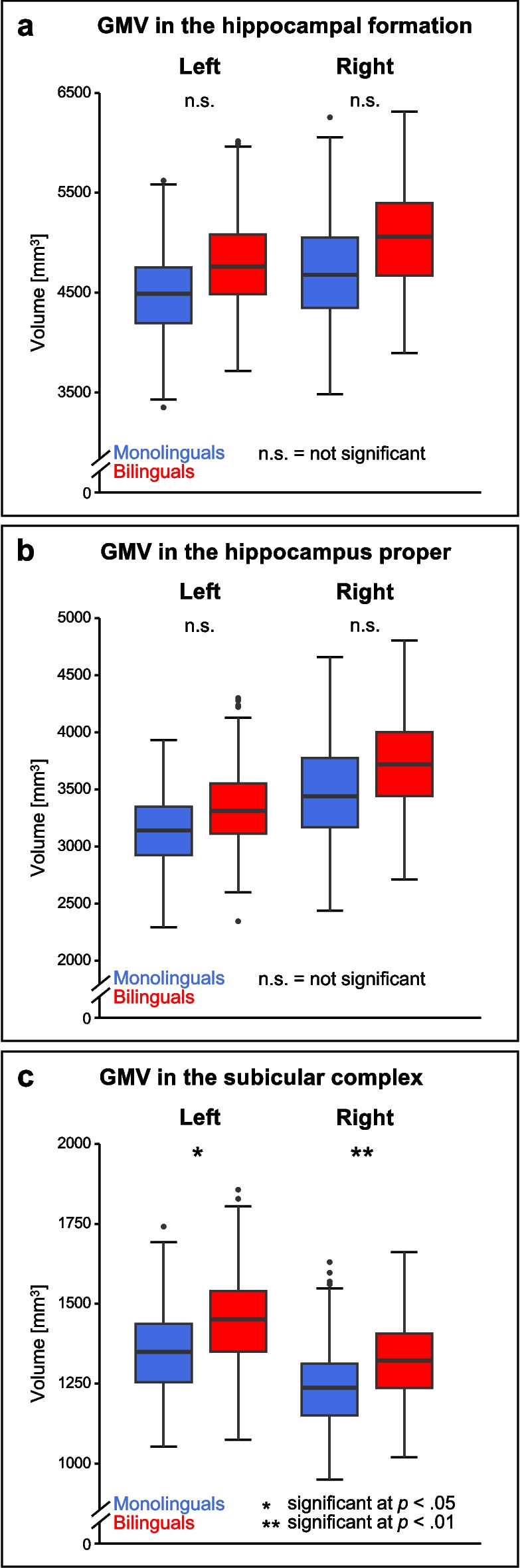
Differences in GMV between mono- and bilinguals of the total sample for the bilateral hippocampal formation (**a**), hippocampus proper (**b**), and subicular complex (**c**). Taking into account the best-fitting model for the relationship between GMV and age within the respective ROI, analyses for the bilateral hippocampal formation and hippocampus proper were based on a quadratic relationship between age and GMV, while analyses for the bilateral subicular complex were based on a linear relationship between age and GMV. Boxplots show median values, lower and upper quartile, maximum and minimum values within the 1.5 interquartile range below/above the lower/upper quartile, and outliers within each language group (please note: these are not outliers with respect to the complete data set). GMV, gray matter volume

Within the total sample, a higher GMV in bilinguals was present on a trend level for the bilateral hippocampal formation (left: *F*(1, 654) = 3.156, *p *= 0.076; right: *F*(1, 654) = 3.432, *p *= 0.064). When investigating hippocampal subregions, however, no GMV difference between language groups could be observed for the bilateral hippocampus proper (left: *F*(1, 654) = 1.645, *p *= 0.200; right: *F*(1, 654) = 1.524, *p *= 0.217), while there was significantly higher GMV in bilinguals within the bilateral subicular complex (left: *F*(1, 655) = 6.403, *p *= 0.012; right: *F*(1, 655) = 10.188, *p *= 0.001; see Fig. 2).

For the older subsample, similar GMV was observed for mono- and bilinguals within the bilateral hippocampal formation (left: *F*(1, 464) = 1.081, *p* = 0.299; right: *F*(1, 464) = 1.937, *p* = 0.165) and the hippocampus proper (left: *F*(1, 464) = 0.356, *p* = 0.551; right: *F*(1, 464) = 0.735, *p* = 0.392). However, there was higher GMV in bilinguals within the right subicular complex (*F*(1, 464) = 6.960, *p* = 0.009), with a trend towards the very same effect within the left subicular complex (*F*(1, 464) = 3.201, *p* = 0.074; see Supplementary Fig. 1).

### Regression analyses

In bilinguals of the total sample as well as the older subsample, regression analyses yielded no effect of factors modulating the bilingual experience on GMV for any of the ROIs (Supplementary Tables 4–5). In contrast, for bilinguals below the age of 55 years, a greater number of languages actively spoken predicted higher GMV in the left hippocampal formation (standardized coefficient *β* = 0.157, *p* = 0.014), while higher bilingual engagement was associated with less GMV in this brain region (standardized coefficient *β* = − 0.231, *p* = 0.002). While the effect for number of languages actively spoken was also found for the left subicular complex (standardized coefficient *β* = 0.194, *p* = 0.003), the effect for bilingual engagement was present for the left hippocampus proper (standardized coefficient *β* = − 0.268, *p* = 0.001). Additionally, higher LoP predicted higher GMV in the left hippocampus proper (standardized coefficient *β* = 0.208, *p* = 0.020). Within the right hemisphere, however, no effect of factors modulating the bilingual experience on GMV was found for the hippocampal formation nor its subregions within bilinguals below the age of 55 years either (Supplementary Table 6).

### Moderation analyses

**Fig. 3:**
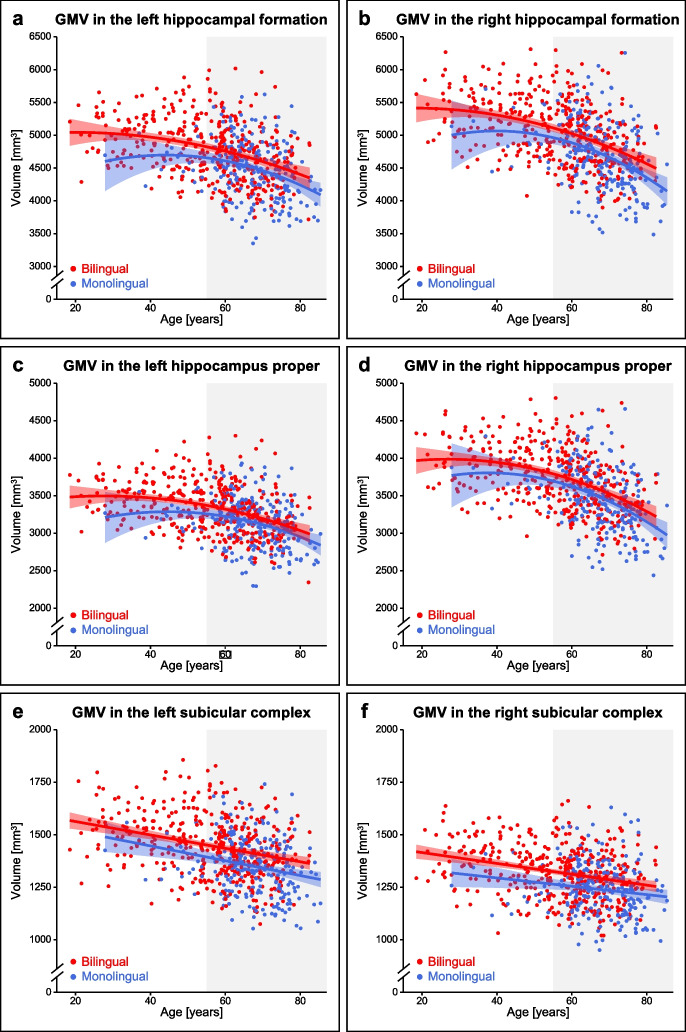
Age-GMV relationships in mono- and bilinguals for the left (**a**) and right hippocampal formation (**b**), left (**c**) and right hippocampus proper (**d**), and left (**e**) and right subicular complex (**f**). Regression lines are depicted with 95% confidence intervals (hippocampal formation and hippocampus proper: quadratic model for the relationship between age and GMV; subicular complex: linear model for the relationship between age and GMV; separately for mono- and bilinguals). The gray underlay indicates participants ≥ 55 years of age, who were included in the older subsample. GMV, gray matter volume

Moderation analyses revealed similar relationships between GMV and age for mono- and bilinguals within all of the analyzed regions for both, the total sample as well as the older subsample (Fig. 3; for model statistics, see Tables 3 and 4). For all ROIs, smaller GMV with higher age was apparent for mono- and bilinguals within both, the total sample as well as the older subsample.

**Table 3:** Results for moderation analyses evaluating the effect of language group (bilinguals = 0, monolinguals = 1) on the relationship between age and GMV and, for the bilateral hippocampal formation and hippocampus proper, age^2^ and GMV for the total sample of 661 participants, including sex (males = 0, females = 1), education, and ICV as covariates

	Hippocampal formation		Hippocampus proper		Subicular complex	
	Left	Right	Left	Right	Left	Right
General model statistics	*R*^2^ = 0.554 *F*(8, 652) = 101.325 *p* < 0.001 ***	*R*^2^ = 0.532 *F*(8, 652) = 92.529 *p* < 0.001 ***	*R*^2^ = 0.483 *F*(8, 652) = 76.059 *p* < 0.001 ***	*R*^2^ = 0.467 *F*(8, 652) = 71.413 *p* < 0.001 ***	*R*^2^ = 0.536 *F*(6, 654) = 126.121 *p* < 0.001 ***	*R*^2^ = 0.529 *F*(6, 654) = 122.264 *p* < 0.001 ***
Moderation effect language group × age	Δ*R*^2^ = 0.001 *F*(1, 652) = 0.757 *p* = 0.385	Δ*R*^2^ = 0.001 *F*(1, 652) = 0.727 *p* = 0.394	Δ*R*^2^ < 0.001 *F*(1, 652) = 0.373 *p* = 0.541	Δ*R*^2^ < 0.001 *F*(1, 652) = 0.177 *p* = 0.674	Δ*R*^2^ = 0.001 *F*(1, 654) = 1.966 *p* = 0.161	Δ*R*^2^ < 0.001 *F*(1, 654) = 0.151 *p* = 0.697
Moderation effect language group × age^2^	Δ*R*^2^ < 0.001 *F*(1, 652) = 0.645 *p* = 0.422	ΔR^2^ = 0.001 *F*(1, 652) = 0.828 *p* = 0.363	Δ*R*^2^ < 0.001 *F*(1, 652) = 0.258 *p* = 0.612	Δ*R*^2^ < 0.001 *F*(1, 652) = 0.230 *p* = 0.631		

*GMV* gray matter volume, *ICV* intracranial volume, ^***^p < 0.001

**Table 4:** Results for moderation analyses evaluating the effect of language group (bilinguals = 0, monolinguals = 1) on the relationship between age and GMV for 470 participants ≥ 55 years, including sex (males = 0, females = 1), education, and ICV as covariates

	Hippocampal formation		Hippocampus proper		Subicular complex	
	Left	Right	Left	Right	Left	Right
General model statistics	*R*^2^ = 0.499 *F*(6, 463) = 76.691 *p* < 0.001 ***	*R*^2^ = 0.442 *F*(6, 463) = 61.197 *p* < 0.001 ***	*R*^2^ = 0.433 *F*(6, 463) = 58.964 *p* < 0.001 ***	*R*^2^ = 0.377 *F*(6, 463) = 46.733 *p* < 0.001 ***	*R*^2^ = 0.489 *F*(6, 463) = 73.926 *p* < 0.001 ***	*R*^2^ = 0.472 *F*(6, 463) = 69.032 *p* < 0.001 ***
Moderation effect language group × age	Δ*R*^2^ < 0.001 *F*(1, 463) = 0.414 *p* = 0.520	Δ*R*^2^ = 0.001 *F*(1, 463) = 1.130 *p* = 0.288	Δ*R*^2^ < 0.001 *F*(1, 463) = 0.017 *p* = 0.897	Δ*R*^2^ < 0.001 *F*(1, 463) = 0.583 *p* = 0.445	Δ*R*^2^ = 0.003 *F*(1, 463) = 2.917 *p* = 0.088	Δ*R*^2^ = 0.003 *F*(1, 463) = 2.659 *p* = 0.104

*GMV* gray matter volume, *ICV* intracranial volume ^***^p < 0.001

### Effects of covariates

Effects of covariates included in the above-described analyses—i.e., sex, education, ICV, and, where applicable, *age* and *age*^*2*^—are reported in Supplementary Tables 4–10.

With sex distribution varying between subsamples (the most unequal distribution of 38.4% females vs. 61.6% males being found in older bilinguals, cf. Table 1), it is worth mentioning that across all analyses, effects of sex were rather small (cf. Supplementary Tables 4–10). While higher GMV in males was observed for the left hippocampal formation, left hippocampus proper, and left subicular complex almost exclusively within the older subsample (cf. Supplementary Tables 4–10), the only ROI for which male sex was significantly associated with higher GMV in almost all of the analyses was the right subicular complex (with *p*-values ranging from *p* = 0.001 in moderation analysis of the total sample to *p* = 0.030 in regression analysis of younger bilinguals; cf. Supplementary Tables 4 and 6–10). The one analysis for which this result could not be obtained was the regression analysis investigating the older bilingual subsample (*p* = 0.157, cf. Supplementary Table 5). In fact, older bilinguals were the only subsample for which no effect of sex was observed in any of the ROIs (cf. Supplementary Table 5).

Post-hoc analyses revealed no interaction for language group × sex for any of the samples within any of the ROIs (cf. Supplementary Tables 11–12).

## Discussion

While previous studies investigated the impact of bilingualism on the whole hippocampal formation, the present study extends this research by specifically also focusing on two distinct hippocampal subregions: the hippocampus proper and the subicular complex. Four major results emerged: (1) Bilinguals within the large total sample showed a tendency towards higher GMV in the bilateral hippocampal formation when compared to monolinguals. This may be interpreted as evidence for higher brain reserve in bilinguals in the hippocampal formation. However, higher GMV in the hippocampal formation was not observed for bilinguals within the older subsample including only participants ≥ 55 years. (2) Investigating subregions of the hippocampal formation revealed contrasting effects: Within the total sample, higher GMV in bilinguals was found for the bilateral subicular complex. For the older subsample, higher GMV in bilinguals was observed within the right subicular complex, while the same tendency was present for its left homologue. Neither for the total sample, nor the older subsample, an effect of bilingualism was found for GMV within the bilateral hippocampus proper. Thus, a differential impact of bilingualism on distinct subregions of the hippocampal formation was observed for the first time, with bilingualism apparently contributing to brain reserve specifically in the subicular complex rather than the hippocampus proper. (3) Factors modulating the bilingual experience, such as AoA, LoP, bilingual engagement, and number of languages actively spoken, did not predict GMV within bilinguals of the total sample, nor of the older subsample, in any of the ROIs. Thus, even though group comparisons had yielded evidence for higher GMV in bilinguals’ bilateral hippocampal formation and subicular complex when compared to monolinguals, evaluating only bilinguals did not reveal a specific additional effect of factors modulating the bilingual experience. When investigating only bilinguals below the age of 55 years, though, a number of actively spoken languages, bilingual engagement, and LoP were revealed to have differential effects on the left hippocampal formation and its subregions. (4) With similar relationships between age and GMV for mono- and bilinguals within the present ROIs, the bilingual brain reserve in the bilateral subicular complex may persist over time.

### The impact of bilingualism on hippocampal structure in older adults

Investigating the impact of lifelong experiences such as bilingualism on structural features of the hippocampal formation and its subregions specifically in older adults entails the challenge of disentangling the effect of the respective experience from the effects of aging. With respect to aging, an inter-individually variable structural brain atrophy has been described [20, 81], particularly pronounced in the hippocampal formation (for reviews, cf. [3, 4]). Structural adaptations to bilingualism have been delineated in the “Dynamic Restructuring Model” (DRM) [72], which will be presented briefly. Subsequently, three hypothetical mechanisms will be discussed that might explain the interaction between bilingualism and aging, ultimately resulting in higher cortical GMV not only in younger, but also in older bilinguals when compared to monolinguals.

The DRM describes three phases of the brain’s adaptations to bilingualism: (1) initial exposure, (2) consolidation, and (3) peak efficiency [72]. As delineated within the DRM, initial exposure to a new language leads to an increase in cortical GMV in regions related to language and executive control, possibly resulting from synaptogenesis, neurogenesis, and/or the formation of novel neural circuits during learning [82]. For the hippocampal formation, this volumetric increase during initial exposure has been demonstrated in language training studies with young adults ([55]: mean age of participants ± SD: 23.61 ± 3.33 years, [56]: mean age of participants ± SD: 20.28 ± 1.44 years). In the light of present results, the hippocampal subregion predominantly showing higher GMV in bilinguals might be the subicular complex rather than the hippocampus proper. The second phase, consolidation, arises with increasing experience in the additional language. Here, a renormalization of cortical GMV is described [72], putatively reflecting a selection of most efficient neural pathways by pruning of inefficient, i.e., under-utilized spines [82, 83]. At the same time, higher subcortical and cerebellar GMV as well as an increase in white matter structural connectivity may be observed, which might represent a shift from lexical acquisition to language control with increasing bilingual experience [72]. For peak efficiency, further adaptations of cerebellar and subcortical GMV and white matter structural connectivity due to increasing bilingual experience and immersion have been described [54, 72].

In relation to the DRM, life-long bilingualism, as investigated in the present study, may correspond to a continuously increasing bilingual experience, possibly resulting in a transition from structural adaptations equivalent to initial exposure, to consolidation, to peak efficiency over time [72]. Evidence for this hypothesis comes from a previous longitudinal study (mean time interval ± SD: 3.6 ± 0.8 years), which found a steeper GMV decline in life-long bilinguals for the left inferior parietal lobule when compared to monolinguals, possibly reflecting continuously increasing bilingual efficiency in these individuals corresponding to the phase of consolidation ([53]; but see the discussion within the paper for alternative interpretations). Thus, in older life-long bilinguals, we would expect similar cortical GMV as in monolinguals, in line with the stages of consolidation and peak efficiency as described in the DRM. Correspondingly, a tendency towards higher GMV in the bilateral hippocampal formation was revealed within the current study only for bilinguals of the total sample, while similar GMV in the hippocampal formation was found for mono- and bilinguals of the older subsample. Additionally, bilinguals of the total sample displayed higher GMV in the bilateral subicular complex, but this effect was only present on a tendency level within the older subsample for the left subicular complex, while remaining significant for its right-hemispheric homologue. At first glance, these findings might indicate a steeper negative age-GMV relationship in bilinguals when compared to monolinguals in the bilateral hippocampal formation and left subicular complex (and one might question why the right subicular complex appears to show a different pattern). However, as moderation analyses revealed similar age-GMV relationships in mono- and bilinguals for both, the bilateral hippocampal formation as well as bilateral subicular complex, caution not to overinterpret these differences in significance for the total sample vs. older subsample (and left vs. right subicular complex) might be advisable. Furthermore, in general, the expectation of similar cortical GMV in older mono- and bilinguals does not seem to be in accordance with results reported in the literature. Higher cortical GMV in regions relevant for language and domain-general control has been found not only in younger, but also in older bilinguals when compared to monolinguals (cf. [72]). With respect to the hippocampal formation, higher GMV has previously been reported in older life-long bilinguals as a function of bilingual exposure [57]. Additionally, investigating hippocampal subregions in the present study provided first evidence that bilingualism might have a differential impact on subregions of the hippocampal formation also in older individuals: while similar GMV was observed for mono- and bilinguals of both samples within the bilateral hippocampus proper, higher GMV in bilinguals’ subicular complex was found not only within the total sample, but also within the older subsample (although this effect was more stable within the right hemisphere).

Higher GMV in older bilinguals may be interpreted in the light of three differential hypotheses: First, bilingualism may modulate the effects of aging on brain structure to the extent that we see a steeper decline in monolinguals when compared to bilinguals, corresponding to the notion of higher brain maintenance [19] in bilinguals. Second, bilinguals may have, e.g., higher cortical GMV to begin with, in accordance with the concept of brain reserve [19]. Thus, older bilinguals would show higher cortical GMV than monolinguals even when experiencing similar age-related decline. Third, bilinguals, especially the ones using their additional languages irregularly, may experience a greater stimulation of synaptogenesis and/or generation of neural pathways than monolinguals even when facing age-related structural decline.

The *first* hypothesis takes into account that higher bilingual GMV might not necessarily reflect increased GMV in bilinguals but a steeper age-related GMV decline in monolinguals [72]—in turn, representing better perseverance of brain structure, i.e., higher brain maintenance [19], in bilinguals. However, to the best of our knowledge, the existing studies reporting differential relationships between age and hippocampal GMV for the two language groups are based on cross-sectional data, while brain maintenance is best assessed longitudinally [19]. Nevertheless, Abutalebi et al. [52] and Li et al. [51] provided evidence for a steeper negative relationship between age and hippocampal GMV for monolinguals when compared to uni- and bimodal bilinguals, respectively. Within the present study, though, no effect of language group on the relationship between age and GMV was evident for any of the ROIs within any of the samples. Notably, in accordance with results from hierarchical regression analyses of the current data, which were in line with previous studies [78, 79, 84], the present study took a quadratic relationship between age and GMV into account, while analyses of both, Abutalebi et al. [52] and Li et al. [51], were based on a linear age-GMV-relationship. Thus, it is possible that cross-sectional analyses based on a linear model for the relationship between age and GMV may overestimate the effect of bilingualism on this relationship within the hippocampal formation and hippocampus proper. With similar age-GMV relationships for the two language groups within all of the ROIs in the present data, it seems questionable whether higher GMV in the hippocampal formation and, specifically, in the subicular complex in bilinguals may truly result from a steeper structural decline in monolinguals.

The *second* hypothesis pertains to the idea that higher cortical GMV putatively acquired by bilinguals in younger ages, e.g., during initial exposure to a new language, may persist over time (cf., e.g., [85]). The present results for the bilateral subicular complex are in line with this assumption: Here, bilinguals showed higher GMV as a proxy for brain reserve as well as similar age-GMV relationships for mono- and bilinguals, possibly resulting in the perseverance of bilingual brain reserve within the bilateral subicular complex well into old age. As the higher GMV in bilinguals was significant for the right subicular complex and found as a tendency for the left subicular complex even within the older subsample for whom no effect of bilingualism on GMV within the bilateral hippocampal formation could be observed, supporting the idea of bilingual brain reserve persistence into old age specifically within the subicular complex, the importance of analyses of subregions when investigating the impact of bilingualism on structural features of the human hippocampal formation becomes evident.

With the bilateral hippocampus proper showing no effect of language group, bilingualism appears to specifically add brain reserve to regions putatively subserving memory retrieval (subicular complex) rather than memory encoding (hippocampus proper) [86–88]. Bilingual brain reserve in the bilateral subicular complex seems particularly beneficial when taking into account that smaller subicular volume has previously been related to a decline in cognition and a higher risk of dementia [17], and atrophy of the subiculum and presubiculum appears to be the earliest hippocampal marker of Alzheimer’s disease [16]. An explanation for this might be a putative decline of the perforant pathway, which penetrates the subiculum to reach the dentate gyrus, as this path has been suggested to be among the first of the brain’s white matter tracts to degenerate in Alzheimer’s disease [16, 89]. Contrastingly, CA1, the largest of the hippocampus proper subfields, rather seems to be affected at later stages of dementia [17].

The *third* hypothesis is based on the notion that one may question whether the unidirectional transition of life-long bilinguals between the three phases of the DRM would take place for bilinguals with highly balanced language use as well as for the ones who do not use their additional language(s) regularly. When second language abilities decline if the respective language is no longer used regularly, a reversion of previous adaptations might occur [90]. Nevertheless, one might hypothesize a re-increase in cortical GMV when using a previously acquired but mostly forgotten language on an interim time period, e.g., a vacation, possibly corresponding to a re-entering of the phase of initial exposure. These “booster-experiences” might therefore result in higher cortical GMV in bilinguals using their languages rather irregularly, possibly stimulating the generation of new dendritic spines and/or neural pathways even in older adults, putatively counteracting age-related structural atrophy (cf. [82]; see also, e.g., [91] for the general possibility of neuroplasticity in older adults).

The three hypotheses putatively explaining higher cortical GMV even in older bilinguals when compared to monolinguals do not mutually exclude one another. Instead, they might describe simultaneous mechanisms reflecting the interactions between aging- and bilingualism-related effects on cortical brain structure. However, in order to disentangle the presumed continuous adaptation to life-long bilingual experience from age-related changes in the older adult population over time, longitudinal studies are necessary.

While higher GMV was found for bilinguals than monolinguals for the bilateral hippocampal formation and subicular complex in dichotomous group comparisons, evaluating the impact of factors modulating the bilingual experience such as AoA, LoP, bilingual engagement, and number of actively spoken languages revealed no additional effect of these parameters on GMV within the hippocampal formation nor its subregions for bilinguals of the total sample, nor of the older subsample. Thus, one might get the impression that bilinguals might not differ substantially from each other in hippocampal GMV as a function of bilingualism-specific parameters. This interpretation would contrast sharply with results reported in the literature, providing evidence for a relationship between hippocampal volume and bilingual exposure [57], bilingual entropy [58], and time spent learning an additional language [55]; additionally, degree of immersion in a second language environment seems related to change with respect to the shape of the hippocampal formation [54]. With these examples, though, it becomes evident that the factors modulating the bilingual experience investigated in the present study do not cover every aspect that may serve to specify the individual bilingual experience. Due to limited data, for example, we refrained from evaluating the impact of immersion in a second language environment in the current analyses (only 71 of the 404 bilinguals of the total sample provided complete data with respect to this parameter; additionally, bilingual immersion was only rather coarsely assessed). While the LEAP-Q prompted participants to indicate whether they spent time in a country, family, school, and/or work environment where the respective second language was spoken, no questions further specifying the experience (such as the extent to which participants used which language in the respective environments, or the amount of time passed since then) were asked. Furthermore, the LEAP-Q, which was used to determine participants’ second language status, did allow to further evaluate current bilingual engagement. However, the impact of some putatively relevant environments, such as partnership(s), school, and work, was not inquired. Thus, a composite variable built from these data would have resulted in an inaccurate proxy for bilingual engagement. Therefore, the parameter “bilingual engagement” used for the above-reported regression analyses was obtained from participants’ indication to which extent on a 5-point scale they were presently confronted with the language for which they had reported the highest LoP, rather than using the more detailed but incomplete data. Hence, future studies may be warranted to examine the influence of differential aspects of bilingual experiences on structural features of the hippocampal formation and its subregions in greater detail.

Nevertheless, an impact of bilingualism-specific parameters was found within the exploratory analyses investigating bilinguals below the age of 55 years. Notably, effects were only observed for the left hippocampal formation and its subregions, which appear preferentially involved in verbal functions, while the right hippocampal formation seems more relevant for the processing of spatial information [92]. Consistent with the notion that acquiring several languages successively may have additive effects on brain structure [72], a greater number of actively spoken languages was related to higher GMV in the left hippocampal formation and the left subicular complex. Higher bilingual engagement was, however, associated with reduced GMV in the left hippocampal formation as well as in the left hippocampus proper. An explanation might be that higher intensity of bilingual engagement may promote neural efficiency, possibly reflected in reduced volumes due to pruning of inefficient synapses (cf. [82]). LoP, on the other hand, might increase with greater knowledge, which may be encoded, for example, in form of new synapses, thus putatively explaining the association between higher GMV in the left hippocampus proper and higher LoP. With age being a very significant predictor of hippocampal GMV in the total sample as well as the older subsample, but not within the younger subsample (an exception being the right subicular complex, showing a reversed pattern), one might hypothesize that the impact of age may override the influence of factors modulating the bilingual experience in older adults, while this may not be the case in younger bilinguals.

### The effect of sex on hippocampal structure in mono- and bilinguals

Sex was originally included in the above-described analyses as a covariate only. Nevertheless, the effects of this parameter on hippocampal GMV within the present sample will be discussed shortly. Higher hippocampal GMV in males, as found in the present data for the left hippocampal formation, left hippocampus proper, and bilateral subicular complex (with effects for the left hemisphere being present almost exclusively in the older subsample, while the effect for the right subicular complex was highly stable across samples and analyses), has previously been reported in the literature (for meta-analysis, cf. [93]; but see also [94] for a meta-analysis that did not find significant differences in hippocampal volume for males vs. females). Future studies may be warranted to investigate potential functional implications of the subregion-specific effects of sex reported here. As no interaction between language group and sex was present, the effect of sex on hippocampal GMV was similar in mono- and bilinguals (and, conversely, the effect of bilingualism was similar in males and females).

### Limitations and future directions

The present study is not without limitations. First, as the included individuals were mostly native German speakers, participants’ second language status was assessed via the pre-existing German version of the LEAP-Q. This approach was preferred over the use of an alternative questionnaire which would have potentially required translation and validation beforehand. However, the LEAP-Q as a self-evaluation tool does not include objective assessment of participants’ language abilities, which might be seen as a disadvantage. Nevertheless, external validity of the LEAP-Q has been shown with respect to objective measures of language abilities [67]. Thus, the LEAP-Q may be considered a sufficient proxy for the evaluation of participants’ language status within the current study.

Second, language families that could be included in the present sample were Germanic, Italic, Balto-Slavic, Hellenic, Uralic, Afro-Asiatic, Austronesian, Japonic, Sino-Tibetan, and Turkic; however, most bilinguals reported abilities in at least two Germanic languages (98.02%), while 51.98% indicated abilities in Germanic and Italic—specifically, Romance—languages, with other language families being comparatively underrepresented (see Table 2). Thus, it is yet to be determined whether the present results would generalize to any combination of languages. Since there seems to exist a common language network across varying language families [95], one might assume some structural brain adaptations to bilingualism to be consistent irrespective of the specific language learned (cf. [62]). Nevertheless, further studies are necessary to determine whether the present results of higher GMV in bilinguals in the bilateral hippocampal formation, and, specifically, in the subicular complex rather than the hippocampus proper, are generalizable to differing language combinations of the same or varying modalities.

Third, the present analyses are based on cross-sectional data only. Thus, we were able to investigate inter-individual GMV differences and differences in age-GMV-relationships in mono- and bilinguals within our ROIs, but we may only hypothesize about intra-individual GMV trajectories over time. With the observation of similar age-GMV relationships in mono- and bilinguals within the hippocampal formation and its subregions in cross-sectional data, we would, correspondingly, expect similar GMV trajectories for the two language groups within these brain regions over time, possibly resulting in the persistence of higher GMV in bilinguals in the subicular complex. To see whether this hypothesis proves true and to further disentangle the effects of aging from the impact of life-long experiences such as bilingualism, longitudinal studies are necessary.

## Conclusion

To the best of our knowledge, the present study is the first large-scale, population-based investigation of structural adaptations to bilingualism not only within the bilateral hippocampal formation, but also within its two subregions, the hippocampus proper and the subicular complex. Importantly, bilinguals’ higher GMV in the hippocampal formation appeared attributable to the subicular complex rather than the hippocampus proper. With similar age-GMV relationships between the two language groups within all of the analyzed ROIs, bilingual brain reserve in the subicular complex may persist over time. This may be particularly beneficial as subicular atrophy has previously been associated with a higher risk for dementia. An influence of factors modulating the bilingual experience such as LoP, bilingual engagement, and number of actively spoken languages has only been observed in bilinguals below the age of 55 years. Altogether, the differential impact of bilingualism on subregions of the hippocampal formation has been demonstrated.

## Supplementary Information

Below is the link to the electronic supplementary material. 

ESM1 (PDF 290 KB)

## Data Availability

The data that support the findings of this study are available from the corresponding author upon reasonable request.

## Footnotes

1 Within the current study, the terms “bilingualism” and “bilinguals” refer to situations where individuals have knowledge of two or *more than* two languages, since a lot of people acquire a third or fourth language over time (cf. [47]).

2 Previous studies investigating the brain’s structural adaptations to bilingualism did not explicitly differentiate between the hippocampal formation and its subregions; in general, they referred to structural features of the “hippocampus.” However, figures illustrating the respective regions of interest suggested that the hippocampal formation as a whole was assessed within these studies. In the following, we therefore refer to results from these studies as pertaining to the hippocampal formation rather than its subregion, the hippocampus proper.

3 Within the present paper, “sex” refers to biological sex. Participants could only choose between “male” or “female.” Gender was not inquired.
