# Supplementary material for: Bilingualism and “brain reserve” in subregions of the hippocampal formation
Source: GeroScience. 2025 Apr 8;47(3):4935–54. doi: 10.1007/s11357-025-01639-0 (PMC12181583; doi:10.1007/s11357-025-01639-0)
Supplement: Supplementary file 1 — (PDF 290 KB) [file 11357_2025_1639_MOESM1_ESM.pdf]

Supplementary Material to

Bilingualism and "brain reserve" in subregions of the hippocampal formation

Katharina Peitz[1,2], Nora Bittner[1,2], Stefan Heim[2,3] & Svenja Caspers[1,2]

1 Institute for Anatomy I, Medical Faculty & University Hospital Düsseldorf, Heinrich Heine University, Düsseldorf, Germany

2 Institute of Neuroscience and Medicine (INM-1), Research Centre Jülich, Jülich, Germany

3 Department of Psychiatry, Psychotherapy and Psychosomatics, Medical Faculty, RWTH Aachen University, Aachen, Germany

Corresponding author:

Katharina Peitz (e-mail: katharina.peitz@hhu.de; k.peitz@fz-juelich.de)

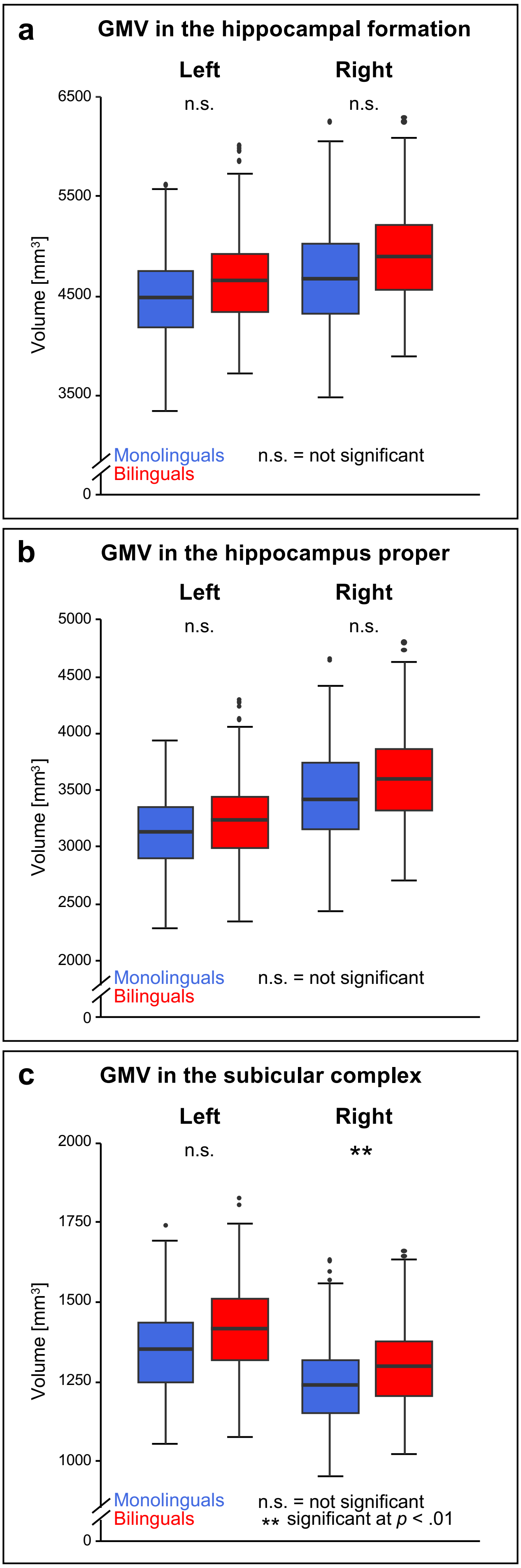

Supplementary Fig. 1 Differences in GMV between mono- and bilinguals of the older subsample including only participants ≥ 55 years old for the bilateral hippocampal formation (a), hippocampus proper (b) and subicular complex (c). All analyses were based on a linear relationship between GMV and age. Boxplots show median values, lower and upper quartile, maximum and minimum values within the 1.5 interquartile range below/above the lower/upper quartile, and outliers within each language group (please note: these are not outliers with respect to the complete data set). GMV, gray matter volume

Supplementary Table 1. Results for hierarchical regression analyses for GMV in the hippocampal formation, hippocampus proper and subicular complex as dependent variable, respectively, for the total sample of 661 participants

	Hippocampal formation		Hippocampus proper		Subicular complex	
	Left	Right	Left	Right	Left	Right
Model 1 – predictor: age	$R^2 = 0.176$ $F(1, 659) = 140.800$ $p < 0.001$ ***	$R^2 = 0.234$ $F(1, 659) = 201.338$ $p < 0.001$ ***	$R^2 = 0.163$ $F(1, 659) = 128.773$ $p < 0.001$ ***	$R^2 = 0.238$ $F(1, 659) = 205.369$ $p < 0.001$ ***	$R^2 = 0.146$ $F(1, 659) = 112.443$ $p < 0.001$ ***	$R^2 = 0.116$ $F(1, 659) = 86.638$ $p < 0.001$ ***
Model 2 – predictors: age, age^2	$R^2 = 0.187$ $F(2, 658) = 75.528$ $p < 0.001$ ***	$R^2 = 0.250$ $F(2, 658) = 109.387$ $p < 0.001$ ***	$R^2 = 0.178$ $F(2, 658) = 71.027$ $p < 0.001$ ***	$R^2 = 0.261$ $F(2, 658) = 116.052$ $p < 0.001$ ***	$R^2 = 0.148$ $F(2, 658) = 57.262$ $p < 0.001$ ***	$R^2 = 0.116$ $F(2, 658) = 43.254$ $p < 0.001$ ***
Significance of ΔR^2	$p = 0.003$ **	$p < 0.001$ ***	$p < 0.001$ ***	$p < 0.001$ ***	$p = 0.166$	$p = 0.984$

GMV gray matter volume, ***p* < 0.01, ****p* < 0.001

Supplementary Table 2. Results for hierarchical regression analyses for GMV in the hippocampal formation, hippocampus proper and subicular complex as dependent variable, respectively, for participants ≥ 55 years old (n = 470)

	Hippocampal formation		Hippocampus proper		Subicular complex	
	Left	Right	Left	Right	Left	Right
Model 1 – predictor: age	$R^2 = 0.104$<br>$F(1, 468) = 54.284$<br>$p < 0.001$ ***	$R^2 = 0.148$<br>$F(1, 468) = 81.000$<br>$p < 0.001$ ***	$R^2 = 0.107$<br>$F(1, 468) = 56.278$<br>$p < 0.001$ ***	$R^2 = 0.166$<br>$F(1, 468) = 92.936$<br>$p < 0.001$ ***	$R^2 = 0.063$<br>$F(1, 468) = 31.607$<br>$p < 0.001$ ***	$R^2 = 0.039$<br>$F(1, 468) = 19.171$<br>$p < 0.001$ ***
Model 2 – predictors: age, age^2	$R^2 = 0.106$<br>$F(2, 467) = 27.603$<br>$p < 0.001$ ***	$R^2 = 0.148$<br>$F(2, 467) = 40.719$<br>$p < 0.001$ ***	$R^2 = 0.109$<br>$F(2, 467) = 28.449$<br>$p < 0.001$ ***	$R^2 = 0.166$<br>$F(2, 467) = 46.480$<br>$p < 0.001$ ***	$R^2 = 0.066$<br>$F(2, 467) = 16.447$<br>$p < 0.001$ ***	$R^2 = 0.043$<br>$F(2, 467) = 10.577$<br>$p < 0.001$ ***
Significance of ΔR^2	$p = 0.335$	$p = 0.470$	$p = 0.417$	$p = 0.667$	$p = 0.260$	$p = 0.164$

GMV gray matter volume, ***$p < 0.001$

Supplementary Table 3. Results for hierarchical regression analyses for GMV in the hippocampal formation, hippocampus proper and subicular complex as dependent variable, respectively, for participants < 55 years old (n = 191)

	Hippocampal formation		Hippocampus proper		Subicular complex	
	Left	Right	Left	Right	Left	Right
Model 1 – predictor: age	R^2 = 0.044<br>F(1, 189) = 8.798<br>*p* = 0.003 **	R^2 = 0.056<br>F(1, 189) = 11.287<br>*p* < 0.001 ***	R^2 = 0.032<br>F(1, 189) = 6.281<br>*p* = 0.013 *	R^2 = 0.037<br>F(1, 189) = 7.174<br>*p* = 0.008 **	R^2 = 0.053<br>F(1, 189) = 10.501<br>*p* = 0.001 **	R^2 = 0.089<br>F(1, 189) = 18.540<br>*p* < 0.001 ***
Model 2 – predictors: age, age^2	R^2 = 0.050<br>F(2, 188) = 4.920<br>*p* = 0.008 **	R^2 = 0.057<br>F(2, 188) = 5.711<br>*p* = 0.004 **	R^2 = 0.035<br>F(2, 188) = 3.438<br>*p* = 0.034 *	R^2 = 0.037<br>F(2, 188) = 3.586<br>*p* = 0.030 *	R^2 = 0.061<br>F(2, 188) = 6.094<br>*p* = 0.003 **	R^2 = 0.094<br>F(2, 188) = 9.783<br>*p* < 0.001 ***
Significance of ΔR^2	*p* = 0.309	*p* = 0.669	*p* = 0.436	*p* = 0.852	*p* = 0.200	*p* = 0.313

GMV gray matter volume, **p* < 0.05, ***p* < 0.01, ****p* < 0.001

Supplementary Table 4. Results for regression analyses for GMV in the hippocampal formation, hippocampus proper and subicular complex as dependent variable, respectively, for bilinguals of the total sample (n = 404)

	Hippocampal formation		Hippocampus proper		Subicular complex	
	Left	Right	Left	Right	Left	Right
General model statistics	$R^2 = 0.547$<br>F(9, 394) = 52.830<br>*p* < 0.001 ***	$R^2 = 0.527$<br>F(9, 394) = 48.769<br>*p* < 0.001 ***	$R^2 = 0.482$<br>F(9, 394) = 40.804<br>*p* < 0.001 ***	$R^2 = 0.458$<br>F(9, 394) = 36.930<br>*p* < 0.001 ***	$R^2 = 0.505$<br>F(8, 395) = 50.355<br>*p* < 0.001 ***	$R^2 = 0.495$<br>F(8, 395) = 48.347<br>*p* < 0.001 ***
**Predictors**	**β**	**β**	**β**	**β**	**β**	**β**
AoA	-0.006	0.003	-0.007	0.012	-0.007	-0.028
LoP	0.051	-0.005	0.079	-0.004	-0.031	-0.003
BiE	-0.028	0.002	-0.038	0.008	0.006	-0.022
NoL	0.018	-0.045	0.006	-0.066	0.045	0.035
Age	0.490 *	0.464 *	0.665 **	0.674 **	-0.298 ***	-0.273 ***
Age^2	-0.829 ***	-0.896 ***	-0.992 ***	-1.112 ***		
Sex (males = 0, females = 1)	-0.023	-0.042	0.003	-0.019	-0.075	-0.105 *
Education	0.056	0.067	0.048	0.075	0.063	0.019
ICV	0.605 ***	0.530 ***	0.568 ***	0.465 ***	0.573 ***	0.570 ***

Models for the bilateral hippocampal formation and bilateral hippocampus proper included *age* and age^2 as covariates to account for the better fit of a quadratic relationship between GMV and age for these regions within the total sample. Models for the bilateral subicular complex included only *age* as a covariate, in correspondence to a linear relationship between age and GMV.

GMV gray matter volume, *AoA* age of acquisition, *LoP* level of proficiency, *BiE* bilingual engagement, *NoL* number of actively spoken languages, *ICV* intracranial volume, *β* standardized coefficient β, **p* < 0.05, ***p* < 0.01, ****p* < 0.001

Supplementary Table 5. Results for regression analyses for GMV in the hippocampal formation, hippocampus proper and subicular complex as dependent variable, respectively, for bilinguals ≥ 55 years old (n = 232)

	Hippocampal formation		Hippocampus proper		Subicular complex	
	Left	Right	Left	Right	Left	Right
General model statistics	$R^2 = 0.518$<br>$F(8, 223) = 29.984$<br>$p < 0.001$ ***	$R^2 = 0.456$<br>$F(8, 223) = 23.348$<br>$p < 0.001$ ***	$R^2 = 0.466$<br>$F(8, 223) = 24.366$<br>$p < 0.001$ ***	$R^2 = 0.\ 397$<br>$F(8, 223) = 18.372$<br>$p < 0.001$ ***	$R^2 = 0.472$<br>$F(8, 223) = 24.944$<br>$p < 0.001$ ***	$R^2 = 0.420$<br>$F(8, 223) = 20.188$<br>$p < 0.001$ ***
**Predictors**	β	β	β	β	β	β
AoA	-0.003	0.008	-0.004	0.024	0.001	-0.044
LoP	0.049	-0.033	0.060	-0.045	0.015	0.015
BiE	0.062	0.052	0.054	0.055	0.070	0.022
NoL	-0.068	-0.075	-0.066	-0.086	-0.061	-0.014
Age	-0.271 ***	-0.334 ***	-0.299 ***	-0.371 ***	-0.153 **	-0.099
Sex (males = 0, females = 1)	-0.038	-0.029	-0.009	-0.006	-0.101	-0.090
Education	0.115 *	0.122 *	0.120 *	0.154 *	0.078	-0.023
ICV	0.582 ***	0.519 ***	0.544 ***	0.441 ***	0.563 ***	0.583 ***

GMV gray matter volume, *AoA* age of acquisition, *LoP* level of proficiency, *BiE* bilingual engagement, *NoL* number of actively spoken languages, *ICV* intracranial volume, *β* standardized coefficient β, *$p < 0.05$, **$p < 0.01$, ***$p < 0.001$

Supplementary Table 6. Results for regression analyses for GMV in the hippocampal formation, hippocampus proper and subicular complex as dependent variable, respectively, for bilinguals < 55 years old (n = 172)

	Hippocampal formation		Hippocampus proper		Subicular complex	
	Left	Right	Left	Right	Left	Right
General model statistics	$R^2 = 0.540$<br>$F(8, 163) = 23.874$<br>$p < 0.001$ ***	$R^2 = 0.482$<br>$F(8, 163) = 18.955$<br>$p < 0.001$ ***	$R^2 = 0.454$<br>$F(8, 163) = 16.920$<br>$p < 0.001$ ***	$R^2 = 0.370$<br>$F(8, 163) = 11.989$<br>$p < 0.001$ ***	$R^2 = 0.511$<br>$F(8, 163) = 21.304$<br>$p < 0.001$ ***	$R^2 = 0.564$<br>$F(8, 163) = 26.392$<br>$p < 0.001$ ***
**Predictors**	β	β	β	β	β	β
AoA	-0.052	-0.035	-0.047	-0.031	-0.052	-0.034
LoP	0.122	0.090	0.208 *	0.128	-0.084	-0.053
BiE	-0.231 **	-0.117	-0.268 **	-0.120	-0.101	-0.069
NoL	0.157 *	-0.014	0.122	-0.056	0.194 **	0.114
Age	-0.112 *	-0.144 *	-0.075	-0.098	-0.165 **	-0.232 ***
Sex (males = 0, females = 1)	-0.002	-0.068	0.027	-0.035	-0.062	-0.143 *
Education	-0.043	0.004	-0.079	-0.027	0.042	0.094
ICV	0.692 ***	0.618 ***	0.645 ***	0.562 ***	0.629 ***	0.583 ***

GMV gray matter volume, *AoA* age of acquisition, *LoP* level of proficiency, *BiE* bilingual engagement, *NoL* number of actively spoken languages, *ICV* intracranial volume, *β* standardized coefficient β, *$p < 0.05$, **$p < 0.01$, ***$p < 0.001$

Supplementary Table 7. Effects of covariates for ANCOVA models for GMV in the hippocampal formation, hippocampus proper and subicular complex as dependent variable, respectively, in the total sample (n = 661)

	Hippocampal formation		Hippocampus proper		Subicular complex	
	Left	Right	Left	Right	Left	Right
Age	$F(1, 654) = 7.035$<br>$p = 0.008$ **	$F(1, 654) = 8.347$<br>$p = 0.004$ **	$F(1, 654) = 8.996$<br>$p = 0.003$ **	$F(1, 654) = 12.160$<br>$p < 0.001$ ***	$F(1, 655) = 101.437$<br>$p < 0.001$ ***	$F(1, 655) = 77.577$<br>$p < 0.001$ ***
Age^2	$F(1, 654) = 21.923$<br>$p < 0.001$ ***	$F(1, 654) = 27.805$<br>$p < 0.001$ ***	$F(1, 654) = 23.657$<br>$p < 0.001$ ***	$F(1, 654) = 33.648$<br>$p < 0.001$ ***		
Sex	$F(1, 654) = 3.706$<br>$p = 0.055$	$F(1, 654) = 3.232$<br>$p = 0.073$	$F(1, 654) = 2.431$<br>$p = 0.119$	$F(1, 654) = 1.288$<br>$p = 0.257$	$F(1, 655) = 3.331$<br>$p = 0.068$	$F(1, 655) = 10.065$<br>$p = 0.002$ **
Education	$F(1, 654) = 1.569$<br>$p = 0.211$	$F(1, 654) = 0.290$<br>$p = 0.590$	$F(1, 654) = 0.516$<br>$p = 0.473$	$F(1, 654) = 0.246$<br>$p = 0.620$	$F(1, 655) = 4.844$<br>$p = 0.028$ *	$F(1, 655) = 0.212$<br>$p = 0.645$
ICV	$F(1, 654) = 283.823$<br>$p < 0.001$ ***	$F(1, 654) = 207.639$<br>$p < 0.001$ ***	$F(1, 654) = 209.163$<br>$p < 0.001$ ***	$F(1, 654) = 139.067$<br>$p < 0.001$ ***	$F(1, 655) = 274.145$<br>$p < 0.001$ ***	$F(1, 655) = 274.515$<br>$p < 0.001$ ***

Parameter estimates revealed that for models including age^2 as a covariate, a significant effect of age^2 reflected less GMV with greater age^2, while a significant effect of *age* corresponded to a positive association between *age* and GMV. For models only including *age*, but not age^2 as a covariate, a significant effect of *age* reflected less GMV with increasing age. Significant effects of education resp. ICV corresponded to higher GMV with higher education resp. ICV. A significant effect of sex reflected higher GMV in males when compared to females.

GMV gray matter volume, *ANCOVA* Analysis of Covariance, *ICV* intracranial volume, *$p < 0.05$, **$p < 0.01$, ***$p < 0.001$

Supplementary Table 8. Effects of covariates for ANCOVA models for GMV in the hippocampal formation, hippocampus proper and subicular complex as dependent variable, respectively, in the older subsample including only participants ≥ 55 years (n = 470)

	Hippocampal formation		Hippocampus proper		Subicular complex	
	Left	Right	Left	Right	Left	Right
Age	$F(1, 464) = 78.303$ $p < 0.001$ ***	$F(1, 464) = 100.280$ $p < 0.001$ ***	$F(1, 464) = 74.328$ $p < 0.001$ ***	$F(1, 464) = 103.803$ $p < 0.001$ ***	$F(1, 464) = 41.324$ $p < 0.001$ ***	$F(1, 464) = 23.152$ $p < 0.001$ ***
Sex	$F(1, 464) = 4.926$ $p = 0.027$ *	$F(1, 464) = 1.890$ $p = 0.170$	$F(1, 464) = 3.925$ $p = 0.048$ *	$F(1, 464) = 0.813$ $p = 0.368$	$F(1, 464) = 4.253$ $p = 0.040$ *	$F(1, 464) = 5.823$ $p = 0.016$ *
Education	$F(1, 464) = 1.679$ $p = 0.196$	$F(1, 464) = 0.436$ $p = 0.509$	$F(1, 464) = 0.868$ $p = 0.352$	$F(1, 464) = 0.690$ $p = 0.407$	$F(1, 464) = 3.109$ $p = 0.078$	$F(1, 464) = 0.059$ $p = 0.808$
ICV	$F(1, 464) = 178.000$ $p < 0.001$ ***	$F(1, 464) = 126.444$ $p < 0.001$ ***	$F(1, 464) = 131.938$ $p < 0.001$ ***	$F(1, 464) = 82.821$ $p < 0.001$ ***	$F(1, 464) = 180.888$ $p < 0.001$ ***	$F(1, 464) = 186.286$ $p < 0.001$ ***

Parameter estimates revealed that a significant effect of *age* reflected less GMV with increasing age. Significant effects of education resp. ICV corresponded to higher GMV with higher education resp. ICV. A significant effect of sex reflected higher GMV in males when compared to females.

GMV gray matter volume, *ANCOVA* Analysis of Covariance, *ICV* intracranial volume, *$p < 0.05$, ***$p < 0.001$

Supplementary Table 9. Effects of covariates for moderation analyses evaluating the effect of language group (bilinguals = 0, monolinguals = 1) on the relationship between age and GMV and, for the bilateral hippocampal formation and hippocampus proper, age^2 and GMV for the total sample of 661 participants

	Hippocampal formation		Hippocampus proper		Subicular complex	
	Left	Right	Left	Right	Left	Right
**Covariates**	β	β	β	β	β	β
Sex (males = 0, females = 1)	-0.062	-0.061	-0.053	-0.042	-0.066 *	-0.107 **
Education	0.040	0.016	0.026	0.016	0.065 *	0.013
ICV	0.546 ***	0.478 ***	0.505 ***	0.418 ***	0.547 ***	0.552 ***

GMV gray matter volume, *ICV* intracranial volume, β standardized coefficient β, *$p < 0.05$, **$p < 0.01$, ***$p < 0.001$

Supplementary Table 10. Effects of covariates for moderation analyses evaluating the effect of language group (bilinguals = 0, monolinguals = 1) on the relationship between age and GMV for 470 participants ≥ 55 years

	Hippocampal formation		Hippocampus proper		Subicular complex	
	Left	Right	Left	Right	Left	Right
**Covariates**	β	β	β	β	β	β
Sex (males = 0, females = 1)	-0.095 *	-0.065	-0.088 *	-0.045	-0.094 *	-0.110 *
Education	0.047	0.021	0.038	0.031	0.059	-0.019
ICV	0.544 ***	0.484 ***	0.498 ***	0.414 ***	0.555 ***	0.572 ***

GMV gray matter volume, *ICV* intracranial volume, β standardized coefficient β, *$p < 0.05$, ***$p < 0.001$

Supplementary Table 11. Post-hoc analyses: ANCOVA models for GMV in the hippocampal formation, hippocampus proper and subicular complex as dependent variable, respectively, to investigate a potential interaction effect for language group × sex within the total sample (n = 661)

	Hippocampal formation		Hippocampus proper		Subicular complex	
	Left	Right	Left	Right	Left	Right
Language group	$F(1, 653) = 3.226$ $p = 0.073$	$F(1, 653) = 3.384$ $p = 0.066$	$F(1, 653) = 1.767$ $p = 0.184$	$F(1, 653) = 1.478$ $p = 0.225$	$F(1, 654) = 6.080$ $p = 0.014$ *	$F(1, 654) = 10.265$ $p = 0.001$ **
Sex	$F(1, 653) = 3.866$ $p = 0.050$ *	$F(1, 653) = 3.022$ $p = 0.083$	$F(1, 653) = 2.850$ $p = 0.092$	$F(1, 653) = 1.132$ $p = 0.288$	$F(1, 654) = 2.643$ $p = 0.104$	$F(1, 654) = 10.098$ $p = 0.002$ **
Language group × sex	$F(1, 653) = 0.166$ $p = 0.683$	$F(1, 653) = 0.021$ $p = 0.885$	$F(1, 653) = 0.713$ $p = 0.399$	$F(1, 653) = 0.075$ $p = 0.785$	$F(1, 654) = 0.891$ $p = 0.345$	$F(1, 654) = 0.107$ $p = 0.743$
Age	$F(1, 653) = 7.114$ $p = 0.008$ **	$F(1, 653) = 8.275$ $p = 0.004$ **	$F(1, 653) = 9.216$ $p = 0.002$ **	$F(1, 653) = 12.023$ $p < 0.001$ ***	$F(1, 654) = 100.017$ $p < 0.001$ ***	$F(1, 654) = 77.549$ $p < 0.001$ ***
Age^2	$F(1, 653) = 22.044$ $p < 0.001$ ***	$F(1, 653) = 27.581$ $p < 0.001$ ***	$F(1, 653) = 24.047$ $p < 0.001$ ***	$F(1, 653) = 33.303$ $p < 0.001$ ***		
Education	$F(1, 653) = 1.501$ $p = 0.221$	$F(1, 653) = 0.298$ $p = 0.585$	$F(1, 653) = 0.443$ $p = 0.506$	$F(1, 653) = 0.261$ $p = 0.610$	$F(1, 654) = 5.069$ $p = 0.025$ *	$F(1, 654) = 0.194$ $p = 0.659$
ICV	$F(1, 653) = 283.591$ $p < 0.001$ ***	$F(1, 653) = 207.240$ $p < 0.001$ ***	$F(1, 653) = 209.350$ $p < 0.001$ ***	$F(1, 653) = 138.765$ $p < 0.001$ ***	$F(1, 654) = 273.614$ $p < 0.001$ ***	$F(1, 654) = 274.239$ $p < 0.001$ ***

A significant effect for language group reflected higher GMV in bilinguals. A significant effect of sex reflected higher GMV in males when compared to females. For models including *age*^{2} as a covariate, a significant effect of *age*^{2} reflected less GMV with greater *age*^{2}, while a significant effect of *age* corresponded to a positive association between *age* and GMV. For models only including *age*, but not *age*^{2} as a covariate, a significant effect of *age* reflected less GMV with increasing age. Significant effects of education resp. ICV corresponded to higher GMV with higher education resp. ICV.

GMV gray matter volume, *ANCOVA* Analysis of Covariance, *ICV* intracranial volume, *$p < 0.05$, **$p < 0.01$, ***$p < 0.001$

Supplementary Table 12. Post-hoc analyses: ANCOVA models for GMV in the hippocampal formation, hippocampus proper and subicular complex as dependent variable, respectively, to investigate a potential interaction effect for language group × sex within the older subsample including only participants ≥ 55 years (n = 470)

	Hippocampal formation		Hippocampus proper		Subicular complex	
	Left	Right	Left	Right	Left	Right
Language group	$F(1, 463) = 0.971$<br>$p = 0.325$	$F(1, 463) = 1.855$<br>$p = 0.174$	$F(1, 463) = 0.350$<br>$p = 0.554$	$F(1, 463) = 0.677$<br>$p = 0.411$	$F(1, 463) = 2.664$<br>$p = 0.103$	$F(1, 463) = 6.954$<br>$p = 0.009$ **
Sex	$F(1, 463) = 4.947$<br>$p = 0.027$ *	$F(1, 463) = 1.894$<br>$p = 0.169$	$F(1, 463) = 3.916$<br>$p = 0.048$ *	$F(1, 463) = 0.818$<br>$p = 0.366$	$F(1, 463) = 4.348$<br>$p = 0.038$ *	$F(1, 463) = 5.796$<br>$p = 0.016$ *
Language group × sex	$F(1, 463) = 0.170$<br>$p = 0.680$	$F(1, 463) = 0.029$<br>$p = 0.864$	$F(1, 463) < 0.001$<br>$p = 0.998$	$F(1, 463) = 0.060$<br>$p = 0.807$	$F(1, 463) = 1.677$<br>$p = 0.196$	$F(1, 463) = 0.027$<br>$p = 0.870$
Age	$F(1, 463) = 77.053$<br>$p < 0.001$ ***	$F(1, 463) = 99.128$<br>$p < 0.001$ ***	$F(1, 463) = 73.673$<br>$p < 0.001$ ***	$F(1, 463) = 102.501$<br>$p < 0.001$ ***	$F(1, 463) = 39.767$<br>$p < 0.001$ ***	$F(1, 463) = 23.078$<br>$p < 0.001$ ***
Education	$F(1, 463) = 1.680$<br>$p = 0.196$	$F(1, 463) = 0.436$<br>$p = 0.509$	$F(1, 463) = 0.866$<br>$p = 0.353$	$F(1, 463) = 0.690$<br>$p = 0.407$	$F(1, 463) = 3.131$<br>$p = 0.077$	$F(1, 463) = 0.059$<br>$p = 0.808$
ICV	$F(1, 463) = 177.778$<br>$p < 0.001$ ***	$F(1, 463) = 126.206$<br>$p < 0.001$ ***	$F(1, 463) = 131.639$<br>$p < 0.001$ ***	$F(1, 463) = 82.691$<br>$p < 0.001$ ***	$F(1, 463) = 181.501$<br>$p < 0.001$ ***	$F(1, 463) = 185.827$<br>$p < 0.001$ ***

A significant effect for language group reflected higher GMV in bilinguals. A significant effect of sex corresponded to higher GMV in males when compared to females. A significant effect of *age* reflected less GMV with increasing age. Significant effects of education resp. ICV reflected higher GMV with higher education resp. ICV.

GMV gray matter volume, *ANCOVA* Analysis of Covariance, *ICV* intracranial volume, *$p < 0.05$, **$p < 0.01$, ***$p < 0.001$
